# Date (*Phoenix dactylifera* L.) by-Products: Chemical Composition, Nutritive Value and Applications in Poultry Nutrition, an Updating Review

**DOI:** 10.3390/ani11041133

**Published:** 2021-04-15

**Authors:** Adel I. Attia, Fayiz M. Reda, Amlan K. Patra, Shaaban S. Elnesr, Youssef A. Attia, Mahmoud Alagawany

**Affiliations:** 1 Poultry Department, Faculty of Agriculture, Zagazig University, Zagazig 44511, Egypt; adelatti159195@gmail.com (A.I.A.); fayizreda@yahoo.com (F.M.R.); 2 Department of Animal Nutrition, West Bengal University of Animal and Fishery Sciences, Belgachia, Kolkata 700037, India; patra_amlan@yahoo.com; 3 Department of Poultry Production, Faculty of Agriculture, Fayoum University, Fayoum 63514, Egypt; ssn00@fayoum.edu.eg; 4 Agriculture Department, Faculty of Environmental Sciences, King Abdulaziz University, Jeddah 21589, Saudi Arabia; 5 The Strategic Center to Kingdom Vision Realization, King Abdulaziz University, Jeddah 21589, Saudi Arabia; 6 Department of Animal and Poultry Production, Faculty of Agriculture, Damanhour University, Damanhour 22516, Egypt

**Keywords:** date by-product, date stone, poultry, nutritive value, production performance

## Abstract

**Simple Summary:**

The demand and price of feed/feed stuffs was greatly altered after the COVID-19 crisis, showing great interest on depends on local feed stuffs and a decrease in the imported ones. Date palm (*Phoenix dactylifera* L.) is a common crop in arid and semiarid regions for production of date fruits. This updating review provides the recent knowledge on the use of date palm by-products and improving their utilization in poultry rations. This article focuses on the chemical composition, amino and fatty acids, mineral content, growth performance, economic aspects and nutritional potential in comparison with widely used corn and barley grains in poultry.

**Abstract:**

Several by-products, such as whole cull dates, date stones (also called pits, seeds, kernels), sugar-extracted date pulp, are produced from date fruit processing industries. These by-products, particularly date stone meal represent 10 to 20% of the whole dates are wasted, causing environmental issues. However, the date stone is rich in various nutrients, such as nitrogen-free extract, fiber, fat, and minerals, which could be used as an alternative feed source in poultry nutrition. However, the high fiber content in date stone meal restricts its use in poultry diets. Whole date wastes and date pulp have lower protein, fiber and fat content than those in date stone meal. Several studies have investigated the use of date stone meal and other by-products as a replacement of dietary corn and barley on feed intake, growth, feed efficiency, and nutrient utilization in poultry. The compilation of results obtained from different studies indicates that date fruit by-products, particularly date stone meal, could be incorporated up to 10% levels, replacing corn or barley grains without hampering production performance, and this could reduce the production cost. Moreover, the use of date stone meal at lower levels (5–10%) sometimes shows better growth performance, probably due to the presence of bioactive principles such as antioxidants and phenolic compounds. The use of date stone meal in poultry diets may be practically important under certain circumstances not only under the COVID-19 crisis due to the lockdown of airports, dry ports, ports and traffic restrictions, but also in countries with a limited supply of classical feed resources and which depend on imported ones. Finally, it can recommend that using date stone meal (DSM) up to 5–10% in poultry diets positively affect the productive performance. Due to the variability in the nutritive value of the DSM, developing a formula for metabolizable energy (ME) of DSM = a (intercept) − b (slope) × crude fiber (CF) content should be considered to improve the precision of feed formula for poultry. However, the nutritional value of DSM might not only depend on crude fiber but also on other compounds.

## 1. Introduction

Several agricultural and agro-industrial by-products are produced during the production or processing of fruits and vegetable crops, which may cause environmental pollution. These by-products were of great interest during the COVID-19 crisis due to lockdown, close of dry borders and ports and traffic restrictions [1,2]. The use of alternative feedstuffs and locally available feed ingredients would improve a possible solution. Date by-products are the best-known by-products that are produced from the arid and desert regions, which are sometimes used in livestock and poultry diets.

In arid and semiarid regions of the world, date palm (*Phoenix dactylifera* L.) constitutes one of the main crops. In countries of the Middle East, date cultivation is common, as it is where about 70% of the world date production occurs [3]. The world date production has been reported to have increased from 1.8 million tons in 1961 to 2.8 million tons in 1985 and to 5.4 million tons in 2001 [4]. The fruit of date palm is comprised of a fleshy pericarp and an inedible seed. A number of date by-products, such as whole cull dates, date stones (also called date pits, seeds, kernels or pips), sugar-extracted fruit date pulp and pressed cakes from date processing are available for date industries [5]. The date stone is a hard oblong body, ventrally grooved and present in the midst of the date fruit, and its length ranges from 12 to 36 mm, while the width ranges from 6 to 14 mm. The weight of the seeds represents 10–20% of the date fruit [6]. The size and weight of the seeds depend upon the variety, maturity and growing conditions. At the back of the seeds, there is a micropyle, where the embryo is located. The embryo is a small oblong body surrounded by the endosperm. The latter is a group of cells inside their walls. The hemicellulose is accumulated, giving it thickness and hardness. Date stone meal (DSM) is rich in carbohydrates and fat along with crude protein content compared to barley and corn grains depending upon the varieties of date palm, which is used as a feed ingredient for livestock feeding. However, DSM contains high amounts of crude fiber, which is the constraint for its use in the diets of monogastric animals, especially for poultry [7]. Tabook et al. [8] pointed out that date fiber can be included at a level of 5% in a broiler diet without affecting the performance of broiler chickens. On the other hand, Slominski [9] reported that enzyme supplementation improved nutrient digestibility and nutrient utilization and allowed use of the agro-industry by-product in poultry nutrition. Moreover, DSM also has functional properties due to the presence of mannans, phenolic compounds and antioxidant activity [5]. A number of studies have been conducted to utilize date fruit by-products as a feed ingredient of poultry diets. The DSM can give a potential alternative for energy source in diet ingredients of poultry. Dietary inclusion of DSM significantly improved the performance and feed utilization of poultry [3,10]. These low-cost feed ingredients have emerged as potential alternatives in poultry that can reduce the cost of rations, but the effects of these by-products on production performance varied among the studies. Therefore, this review was undertaken to compile the findings of the experiments on date fruit by-products for the chemical composition, nutritive values, growth performance, carcass traits and economic aspects in comparison with widely used corn and barley grains in poultry.

## 2. Chemical Composition and Feeding Values of Date Waste

### 2.1. Proximate Analysis

The proximate analysis of DSM was studied by several investigators and is shown in Table 1. The chemical composition (%) ranges widely between 88.2 and 92.0, 5.27 and 10.5, 2.89 and 9.43, 8.22 and 28.9, 58.0 and 72.4 and 0.98 and 9.08 for dry matter (DM), crude protein (CP), ether extract (EE), crude fiber (CF), nitrogen free extract (NFE) and ash, respectively. Additionally, the DSM contains 3.3 MJ/kg DM true metabolizable energy (TME) for poultry. Based on the chemical composition of DM, the nutritional value is very low for TME, due to the very high fiber content and the low protein content, resulting in metabolizable energy levels as low as 2.9 MJ/kg. It can be used in poultry diets provided that this low energy level is taken into account in diet formulation and compensated by supplementation of oil or fat [11].

The chemical composition of dried date press cake (excluding the pits) varies, particularly for CF, CP and NFE depending upon the sources and varieties of dates but are within the range of 87.7 to 95.2 for DM, 4.4 to 8.1 for CP, 9.1 to 21.8 for CF, 1.8 to 2.7 for EE, 2 to 3.5 for ash and 55.3 to 72.7 for NFE on a dry matter basis [5]. The chemical composition of date wastes is usually close to the chemical composition of corn and barley, except for CF values which are higher than those of corn and barley (8.22–16.6 vs. 2.3 and 5.0%, respectively). Higher fiber content is present in DSM or whole date fruit due to the presence of greater fiber content in the outer layer of the date seeds. The pulp of the dates has less fiber, low CP and higher NFE. The sugar extracted date fruit pulp was reported to contain 5.5% CP, 11.8% CF, 0.4% EE, 79.6% NFE and 2.7% ash [12]. Thus, pulp can be added to broiler diets at levels up to 20%. As date pulp contains less CP, whole date wastes have a low CP (2.9%), which is much lower than for DSM, maize and barley [13]. The fruit flesh is rich in NFE (76.2%), which is closer to values of barley (79.0%) but greater than that of DSM. The amino acid in flesh, mainly methionine (0.06%) and lysine (0.10%) are lower than those in the DSM, maize and barley due to lower CP concentration in the flesh [14].

Date is found to be low in protein and fat but highly rich in sugars, especially glucose and fructose [15]. Date is a good source of energy, where 100 g of date can provide about 1.31 MJ. In addition, minerals were reported, such as selenium, potassium, copper, and magnesium. The daily consumption of 100 g can cover about 15% of the recommended requirement of these minerals. Date contains about 8.0 g/100 g date, but insoluble dietary fiber is the major part of dietary fiber in this fruit. Regarding the vitamin content, vitamins C and B-complex are the major vitamins in dates. It is also a good source of antioxidants, mainly phenolic compounds and carotenoids. The seeds of date contain considerable concentrations of fat (9.0 g/100 g) and protein (5.1 g/100 g) when compared to the flesh. Additionally, seeds are high in phenolics (3942 mg/100 g), dietary fiber (73.1 g/100 g), and antioxidants (80,400 mmol/100 g) [16].

Shaba et al. [17] reported that date palm fruits contain low amounts of anti-nutritional factors, such as oxalate, tannin, saponin, alkaloid and cyanide. This indicates that the date seeds can be used effectively as the anti-nutritional composition is low and there would be no interference with the nutrient-like minerals and protein in the body. Depending on different varieties of date by-products, date pits contain significant amounts of micronutrients and macronutrients, but all varieties are excellent sources of fiber and maybe used as important sources of functional foods [18,19,20].

### 2.2. Digestion Coefficients and Feeding Values of Date Waste

The digestibility values of CP, CF and NFE, in general, were lower than those of barley and corn, while the digestion coefficient of EE was better than those of barley and corn (Table 2). Metabolic energy values of DSM were lower than that of corn (14.14 MJ/kg). More studies in this regard revealed the nutritional value of DSM. For example, Sawaya et al. [23] reported that the protein digestibility and calculated protein efficiency ratios were 64.1, 1.67 for Ruzeiz, 62.9, 1.65 for Sifri date, respectively, with an average of 63.5, and 1.65 for Ruzeiz and Sifri compared to 90.0 and 2.50% for casein. Attia and Al-Harthi [12] evaluated date waste in broiler chickens fed at 0 to 200 g/kg diet replacing wheat bran containing similar energy and protein concentration and observed that digestibility of nutrients was not affected except decreased CP digestibility at 200 g/kg. In addition, El-Deek et al. [24] reported that inclusion (0 to 150 g/kg diet at 6 levels) of inedible date waste (85% fruit and 15% pits) in broiler chickens did not affect nutrient digestibility. Al-Homidan [3] reported ME values of 7.23 and 4.71 MJ/kg, respectively, for broiler chickens. In future, developing a formula for ME of DSM = a − b × CF seems essential due to variability in the nutritive value of DSM to improve the accuracy of feed formula for poultry. Nonetheless, the nutritional value might not only depend on crude fiber but also on other compounds.

### 2.3. Fiber Fractions

Fiber fractions of DSM as reported by several workers, compared with yellow corn and barley, are shown in Table 3. The values ranged from 39.6 to 57.5, 51.6 to 75.0, 12.0 to 17.5, 26.1 to 42.5 and 7.21 to 11.0 for acid detergent fiber (ADF), neutral detergent fiber (NDF), hemicellulose, cellulose, and lignin, respectively, which were higher than those of both corn and barley. Fiber fractions of DSM were reported to be 57.6, 46.4 and 13.7 for NDF, ADF and ADL, respectively [25]. These results indicated that the low-energy values of DSM compared with corn and barley could be due to higher fiber fractions [11].

### 2.4. Fatty Acid Content

Oil extracted from date seeds is a pale yellowish–green in color and has a pleasant odor [5,26,27]. Abdel-Nabey [19] reported that the crude oil extracted from date pits has a pale-yellow color and is semi-solid at refrigerated temperature (−40 °C). The main median characteristics are as follows: an acidity value of 1.11, 0.96% unsaponifiable matter, a refracted index of 1.46, an iodine value of 49.7 and a saponification value of 219.6. The date seed oil is a natural source of medium-chain fatty acids (MCFAs) such as lauric acid (10.36–35.31%) and capric acid (0.2–0.8%). These MCFAs are effective against a wide range of microorganisms in chicken intestines and can improve the growth performance and immunity of poultry [28].

The values (% of fat) ranged between 12.20 and 23.06, 9.70 and 11.30, 10.11 and 12.70, 1.56 and 3.56, 35.1 and 45.80, 8.10 and 11.00, and 0.37 and 0.80 for lauric, myristic, palmitic, stearic, oleic, linoleic and linolenic acids, respectively. The values were close to the corn, except for myristic and oleic fatty acids, which were higher than those of corn were. Corn contained higher linoleic and linolenic than DSM (51.5 and 1.7% vs. 8.10 to 11.0% and 0.08 to 0.37%, respectively). Abdel-Nabey [19] reported that date pit oil has a very low content of linolenic acid (0.37) in comparison with many vegetable oils, i.e., cottonseed, sunflower and maize oils. The ratio of unsaturated to saturated fatty acids varies from 1.10 to 1.20 with an average of 1.17 because the total amount of unsaturated fatty acids is slightly higher than those of saturated fatty acids. This ratio, however, is lower than those of the common edible vegetable oils in which their degree of unsaturation is quite high.

### 2.5. Amino Acid Content

Amino acid content of DSM as indicated by several studies, compared to corn and barley, are shown in Table 4. The highest amino acid contents of DSM are almost close to those of corn grain, while they are lower than those of barley, except for arginine which is higher than that of corn and close to that of barley (0.36–0.60 vs. 0.17 and 0.52%).

### 2.6. Mineral Content

Studies showing both major and trace elements of DSM in comparison to corn and barley are presented in Table 5 and Table 6. Date stone meal contents of Mg, K and Na are close to those of both corn and barley. The level of P is close to that of corn, but lower than that of barley, while the content of Ca is higher than those of both corn and barley. The trace elements (Zn, Cu, Mn and Fe) of DSM are in general higher than those of corn but close to those of barley. Calcium and phosphorus levels (0.76 and 0.52%, respectively) are present in higher amounts than in maize and barley. The protein content (8.1%) in DSM is higher than that of whole date but is nearly close to that of maize grains (8.6%) [14].

## 3. Effect of Date Waste on Poultry

### 3.1. Effect of Date Waste on Growth Performance

Several studies recommended that DSM up to 10% in broiler diets can be incorporated without negative effects on growth performance [1,4]. El-Deek et al. [24] tested six levels (0 to 150 g/kg diet) of inedible date waste (85% fruit and 15% pits) in broiler chickens and noted that body weight, feed intake and feed efficiency were similar at all levels of date waste. Date palm pollen is used to improve fertility and ovulation in men and women due to it contains fatty acids, amino acids, saponins, flavonoids and sterols. Egg mass, egg weight and egg production of laying hens treated with different levels of date palm pollen were increased when compared to the control [32]. Jaffer and Jassim [33] used 0, 5, 10 and 15 of % date by-product with or without enzyme instead of maize and they found body weight, feed intake, and feed conversion as well as economic feasibility were not affected by this by-product.

As DSM contains high fiber content, which limits its use in poultry diets, some processing of DSM has been performed to improve feed utilization efficiency and performance of chickens. For example, Al-Saffar et al. [34] conducted two experiments to investigate the effect of phytase and/or multienzymes (xylanase, β-glucanase, cellulase, hemicellulose, protease and amyloglucosidase) on improving the utilization of DSM in laying hens. In experiment 1, DSM completely replaced corn in isocaloric and isonitrogenous diets, but with added phytase (500, units of phytase (FTU)/kg) and/or multienzymes (0.1%). In experiment 2, DSM was added at 0, 15, and 30% in isocaloric and isonitrogenous diets, and diets containing DSM were fed without or with phytase and/or 0.1% multienzymes. In experiment 1, laying performance and eggshell quality of hens reduced significantly due to complete substitution of corn, but hatchability and fertility were not influenced. Supplementation of phytase and multienzymes did not restore the laying performance compared to the control level. In experiment 2, the results showed that DSM could be included in diets up to 30% when supplemented with multienzymes without affecting the laying performance of hens.

Hussein and Alhadrami [35] used date pits at 0, 10, 15, and 20% with or without enzyme supplementation (Avizyme 1500) to the starter and finisher diets, while grower diets contained 10% uncooked or cooked date pits. The results showed that adding date pits with or without enzyme to the starter and finisher broiler diet had no effect on broiler performance. Body weight gain of birds who received 10% uncooked date pits were significantly higher compared to the control group birds. Groups fed a diet containing 10% date pits had no effects on feed intake or feed conversion ratio. Barreveld [5] reported that date stone could successfully replace 10% barley in chick diets. The increased live gain over the control is related to more feed intake and may be due to the existence of a growth promoting substance in date stone. Similarly, date waste (0 to 200 g/kg diet replacing wheat bran containing similar energy and protein concentration) fed to broiler chickens did not affect body weight gain up to 200 g/kg diet [36].

Al-Homidan [3] determined the effect of adding date waste (whole date waste meal and date pits meal) in starter and finisher broiler diets. Seven levels of date waste representing 0, 8, 16, 24% date waste meal and 5, 10, and 15% date pits meal were used in the starting period, while 0, 8, 16, 24% date waste meal and 6, 12 and 18% date pits meal were used during the finishing period. Results indicated that body weight and daily gain were improved when chicks were fed diets containing 8% date waste meal or 10% date pits meal during the starting period (0–4 weeks). During the finishing period (5–7 weeks), chicks fed a diet containing 8% and 16 date waste meal or 18% date pits meal had better body weight and daily gain than the control. Feed conversion improved significantly when the level of date pits meal was 10% during the growing period. During the finishing period, feed conversion was better when diets included 18% date pits meal compared with the control.

In broiler chickens, date waste (0 to 200 g/kg diet replacing wheat bran) reduced feed intake and improved feed efficiency and European feed production efficiency at 50 g/kg, whereas these variables in other date waste levels were similar to the control [12]. Kamel et al. [14] fed diets containing date pits at 5, 10 and 15%, and whole zahdi dates at 5, 10, 30 and 47.7% to broiler chicks. They found that the incorporation of 47.7% whole date as a total replacement of corn resulted in a slight decrease in feed conversion of chicks.

In some studies, the use of DSM improved production performance when used at low levels compared with the corn or barley grains, which is attributed to the presence of bioactive compounds present in it [34,35,36,37]. In addition, DSM may be effective against the aflatoxicosis. Abdel-Sattar et al. [36] reported that the inclusion of DSM (2 and 4%) in diets of broiler chickens ameliorated the negative effects of aflatoxins and provided a partial protective effect against aflatoxicosis, which was dose-related as 2% supplementation provided better protection than 4% supplementation. Moreover, supplementation of DPS in the diets reduced malondialdehyde content in muscle.

El-Far et al. [37] conducted a study on broiler chickens fed diets containing 2, 4 and 6% DSM. Results indicated that DSM significantly increased serum reduced glutathione content for all DSM, and interferon-gamma and interleukin-2 levels were significantly higher in chicks fed 2% DSM. Therefore, the authors concluded that increase growth performance, immunity, and antioxidant status of chickens due to DSM supplementation might be related to the antioxidant and immune-stimulant constituents in DSM.

The productivity and qualitative traits of eggs of laying hens were not adversely affected by the inclusion of date pits in the diets [38]. Hermes and Al-Homidan [39] clarified that the best performance was seen when 16% date waste meal or 10% date pits meal could be replaced by yellow corn without any adverse effect on productive performance, egg quality. Up to 20% date seeds was tested without an adverse effect on egg production and feed efficiency, but it decreased egg quality [40]. Al-Harthi et al. [41] recommended that using date pits as alternative feedstuff enhanced maturity of Lohmann pullets and reproduction performance of layers. Ghasemi et al. [42] stated that date pits at the level of 10% could be partly used as alternative feedstuffs in laying hens’ diets, without negatively affecting the productive and health parameters but more than 10% feed intake was reduced due to the presence of non-starch polysaccharides which increase the viscosity of gut contents.

Generally, the age of the bird affects the benefit from adding dates to the diets, as old birds benefit from the fibers more than young birds. Due to the high percentage of fiber in date wastes, it should be added to finisher diets with a higher percentage than the starter diets. Additionally, ducks and laying hens benefit from the diets containing date waste more than broiler chickens. Finally, from the previous studies that were reviewed, it can be concluded that using DSM up to 5–10% in diets of poultry (broiler, laying hen, ducks, quail or turkey) positively affects the productive performance. We summarized effects of date by-products on performance of poultry in Table 7.

### 3.2. Effect of Date Waste on Carcass Traits

Kamel et al. [14] fed broiler chicks with diets containing date pits at 0, 5, 10 and 15% or whole Zahdi dates at 0, 5, 10, 30 and 47.7%. The gross examination of various internal organs (liver, heart, spleen, pancreas and intestines) in both date by-products revealed abnormalities. The weight of inedible parts (including feather) of carcass as well as their proportional weight showed an insignificant response to the effect of date seeds levels [4]. The absolute and percentage weights of total edible meat differed significantly between 5% date seeds (1188 g) and the control group (1347 g). Differences for carcass weight between the 5% group (1077 g) and the control group (1229 g) were also significant, while differences between 5% and other groups were not significant. Percentage of carcass weights of the control group (66.4%) was higher than the other groups except 10% (67.2%). In addition, giblet weights were not significant for all groups.

Osman et al. [44] studied the extent of using DSM in starter and grower Pekin duckling diets. The results indicated that dressing percentage was not significantly affected by the level of DSM in the diets, but gizzard percentage increased when increasing the level of DSM in the diets. Moreover, there were pronounced trends for increased absolute and relative digestive tract weight and length of digestive tract and caecum when increasing level of DSM in the diets. This could be attributed to the gritty nature and to the high fiber content of DSM. Such increase in gizzard percentages may be an adaptation of the birds to digest the gritty and fibrous feed at the high levels of DSM. In addition, both small intestine and caecum length increased gradually with the increasing of DSM, but fasting body weight, carcass, liver and heart percentages were not affected by the level of DSM in the broiler diets.

Al-Homidan [3] determined the effect of addition of date wastes (whole date waste meal, and date pits meal) to starter and finisher broiler diets containing 0, 8, 16 and 24% whole date waste meal, or 5, 10 and 15% date pit meal in the starting period and 0, 8, 16 and 24% whole date waste meal and 6, 12 and 18% date pit meal during finishing period. Results indicated that the corresponding values for dressing percentage ranged between 68.7 and 70.5%, while ready to cook (hot carcass weight and giblets weight) percentages ranged between 73.3 and 75.6%, without significant differences among the levels.

Attia and Al-Harthi [12] reported that date waste in broiler chickens fed at 0 to 200 g/kg diet did not affect dressing percentage, weight (%) of liver, gizzard, but altered many other carcass traits such as weight (%) of heart, spleen, pancreas and intestine. Similarly, inedible date waste up to 150 g/kg diet of broiler chickens did not affect dressing percentage, chemical composition of meat along with water holding capacity, pH, tenderness, and color, but weight (%) of heart, pancreas and spleen differed among the inclusion levels [37].

### 3.3. Effect of Date Waste on Mortality

Regarding mortality, Kamel et al. [14] found no effect due to DSM levels on broiler mortality. They reported that there was no link between chick mortality and the use of date pits in the diets. The effect of using DSM as a substitute for yellow corn in chickens quail diets up to 24% levels on the mortality rate of Japanese quails was studied [36]. These studies reported that up to 10% DSM can be fed to chickens without affecting growth performance. Al-Homidan [3] found no significant effect on broiler mortality rate (the mortality rate was 2.2% during total experimental period of 0 to 7 weeks) due to using date waste (0, 8, 16, 24% whole date waste meal) or (5, 10, and 15% DSM) during the starter period, or (0, 8, 16, and 24% whole date waste meal) and (6, 12 and 18% DSM) during the finishing period. Date waste up to 200 g/kg diet replacing wheat bran did not affect mortality of chickens [37].

### 3.4. Effect of Date Waste on Feed Cost and Economic Efficiency

The net profits in broilers fed 0%, 1%, 2%, 3% and 4% of date palm kernel were 27.01, 32.77, 36.78, 43.47 and 44.51 (Rs.) per broiler, respectively. It was reported that the maximum net profit (44.51/bird) was recorded from the birds fed 4.0% date palm kernel, closely followed by those birds fed 3.0% date palm kernel (43.47/bird) [45]. Furthermore, in broilers, the feed cost per kg live weight gain was statistically less in birds fed date palm kernel with or without enzymes compared to the basal diet [46].

**Figure 1:**
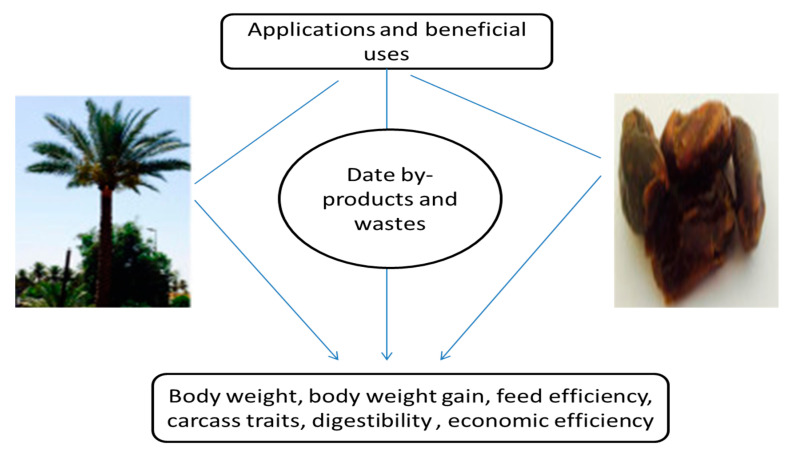
Application and beneficial uses of date by-products and wastes in poultry nutrition.

Al-Homidan [3] found that using date wastes (whole date waste meal and DSM) at 5, 10 and 15% in broiler diets reduced the cost of the diet compared with the control group. Application and beneficial uses of date by-product and wastes in poultry nutrition are illustrated in Figure 1.

## 4. Recent Knowledge and Future Direction

Recently, there have been trends to utilize the phytochemical properties of industrial fruit or herbs wastes in animal feeding, which will not only reduce environmental burdens, but also improve animal production and health [47,48]. Similarly, phytochemical or nutraceutical prosperities of date pit or waste have been explored or attempts have been made to improve them using solid state degradation by *Trichoderma reesei* L. and their utilization in animal feeds [49,50,51,52,53]. Alyileili et al. [49] reported that solid state degradation of date pit with *T. reesei* enhanced mannan oligosaccharide content, phenolic (142 g gallic equivalent/kg) and flavonoid (117 g rutin equivalent/kg) content of degraded date pits and antioxidant activities. Due to the presence of probiotic, phenolics and flavonoids along with high antioxidant activities, the degraded date pits might provide beneficial impacts on gut health and barrier function [54,55]. Therefore, extra-nutritional effects of *T. reesei*-mediated solid state degraded date pit were explored in poultry [56,57]. The inclusion of 10% degraded date pits (phenolics, flavonoids and mannan–oligosaccharide) in the diets of broiler chickens increased catalase, superoxide dismutase and glutathione peroxidase activities in the serum, liver, and intestine, and decreased malondialdehyde content compared to the control group [50]. It also increased pancreatic enzyme activities, the villus length, and the villus/crypt ratio, and decreased the crypt depth of the intestine [51]. The number of pathogenic bacteria such as *Escherichia coli,* *Enterobacteriacea, Salmonella*, and *Shigella* were reduced, but beneficial *bacteria such as Bifidobacterium* and *Lactobacillus* were increased in the gut of poultry by 10% degraded date pits [52]. Additionally, immunoglobulin A and G content in serum and intestinal tissue, gene expressions of mucin-2, cathelicidin-1, beta defensins, and liver expressed antimicrobial peptide-2 (which prevents the attachment of invading pathogens in intestinal epithelia) in jejunum of chickens fed degraded date pits were increased [52]. However, body weight, feed intake, and feed conversion ratio were not influenced by 10% degraded date pits compared with the control [52]. Alyileili et al. [53] compared 5 and 10% non-degraded date pits vs. degraded date pits in chickens. The populations of *Escherichia coli, Salmonella*, *Campylobacter* and *Shigella* spp. were reduced in both degraded and non-degraded date pit-diets compared with the control, but degraded date pit diets had a stronger effect than the non-degraded date pit diets. Body weight gains were not affected, but feed conversion ratio was higher for non-degraded date pits diets than control and degraded date pit diets. European production efficiency index was greater with 5% and 10% degraded date pits than non-degraded date pits and the control [53]. Overall, these results suggested that date pits can improve gut health by reducing pathogenic bacteria, enhancing beneficial bacteria, gene expressions of antimicrobial peptides in the jejunum, and immunoglobulin concentrations in the intestine and serum while degraded date pits likely have greater effects than the non-degraded date pits. Future studies may also investigate the gut barrier and electrophysiological functions such as mRNA expressions of tight junction proteins, permeability, especially during a pathogenic microbial challenge models due to the presence of plant bioactive principles (such as phenolics and flavonoids) in non-degraded or degraded date pits because these plants bioactive have been shown to improve the gut barrier function in animals including in poultry [56,57].

Dates by-products are currently used in the feeding of animals, poultry and fish industries. Due to the presence of a large amount of total fiber, they are considered to have potential health benefits for human as prebiotics [58]. The dietary fiber concentrate of date seeds/kernels presented the potential to be used as a novel source of prebiotic feed additives, by increasing the numbers of *Lactobacillus paracasei* as probiotics [58]. Fiber concentrate of date fruit has been converted into another product rich in antioxidant soluble fiber by enzymatic hydrolysis [59].

Based on the nutritional values and chemical composition of the protein, carbohydrate, fat and minerals content in the date kernels, it has been reported that they could be used as a novel source of functional products with interesting technological functionalities that could also be applied as an excellent source of prebiotic due to the high content of dietary fiber [16,60]. All the above information will enhance the value of date by-products, fruits, seeds, etc. as excellent and cheap sources of a natural diet that can act as bioactive and nutritive ingredients in the pharmaceutical industries, food sector, and for other aspects [61,62].

## 5. Conclusions

Date fruit by-products, particularly DSM, have good nutritional values in poultry diets. The date has high concentrations of oleic acid and lysine and methionine content which are comparable to the corn and barley grains. Considering variability in the nutritive value of the DSM, developing a formula for ME of DSM = a (intercept) − b (slope) × crude fiber content should be considered to improve the feed formula accuracy. However, the nutritional value might not only depend on crude fiber but also on other compounds.

The results obtained from different studies indicate that date fruit by-products, particularly DSM, could be incorporated up to 5–10% levels, replacing corn or barley grains without hampering feed intake, growth, feed efficiency and nutrient utilization in poultry (broiler, laying hen, ducks, quail and turkey), and this could reduce the production cost. Maximum levels of DMS should be 5% in young birds and 10% in grower–finishers. Higher levels could be acceptable in slow-growing birds due to low-nutritional requirements. It also seems that young birds are less tolerant of DSM than older birds. In layers, up to 20% DMS was evaluated without any adverse effect on feed efficiency and egg production, but it decreased egg quality. The balance of protein and energy in the diet should always be established carefully. These agriculture-by-products become important if circularity becomes more important, not only in the Covid-19 crisis, but also in countries with a limited supply of classical feed resources and dependence on imported ones.

## Figures and Tables

**Table 1:** Chemical composition (%) of different varieties of date stone meal compared with yellow corn and barley.

References	Variety	DM	CP	EE	CF	NFE	Ash	TME (MJ/kg DM)
[19]	Sewi	91.4	6.44	8.16	14.2	70.2	0.98	ND
[21]	ND	90.9	10.5	9.43	8.60	69.5	2.01	ND
[22]	ND	90.5	6.4	6.70	28.9	58.0	1.9	3.30
Yellow corn *	-	88.0	7.7	3.8	2.3	84.8	1.4	ND
Barley *	-	88.0	11.9	2.00	5.00	78.8	2.3	ND

* Central laboratory of food and feed, CLFF; ND: not defined or not determined; DM = dry matter; CP = crude protein; EE = ether extract; CF = crude fiber; NFE = nitrogen free extract, TME = true metabolizable energy.

**Table 2:** Digestion coefficients (%) and metabolizable energy (ME, MJ/kg) of different varieties of date stone meal compared with yellow corn and barley.

References	Species	Variety	DM	OM	CP	CF	EE	NFE	ME, MJ/kg
[14]	Broiler	Sewi	45.0	44.2	42.9	3.45	84.2	49.7	9.57

DM = dry matter; OM = organic matter; CP = crude protein; EE = ether extract; CF = crude fiber; NFE = nitrogen free extract; ME = metabolizable energy.

**Table 3:** Fiber fractions (% of dry matter) of date stone meal compared with yellow corn and barley.

Fiber Fraction	Date Stone Meal [5]	Yellow Corn ***	Barley ***
ADF *	57.5	2.20	6.00
NDF **	75.0	9.00	17.0
Hemicellulose	17.5	ND	ND
Cellulose	42.5	2.00	4.00
Lignin	11.0	1.00	2.00

* Acid detergent fiber (cellulose + lignin); ** Neutral detergent fiber (hemicellulose + cellulose + lignin); *** Central Laboratory of food, CLFF [22]; ND: Not defined or not determined.

**Table 4:** Amino acid content (% of dry matter) of date stone meal compared with yellow corn and barley.

Amino Acid	[29]	[30]
	Date Stone Meal	Barley
Aspartic	0.52	ND
Threonine	0.17	0.37
Serine	0.20	0.46
Glutamic	0.98	ND
Proline	0.19	ND
Glycine	0.27	0.44
Alanine	0.25	ND
Valine	0.28	0.52
Methionine	0.09	0.18
Isoleucine	0.20	0.37
Leucine	0.33	0.76
Tyrosine	0.06	0.35
Phenylalanine	0.20	0.56
Histidine	0.12	0.27
Lysine	0.27	0.40
Arginine	0.60	0.52
Cystine	0.10	0.24
Protein	5.70	11.0

ND: not determined.

**Table 5:** The major mineral element content (% of dry matter) in different varieties date stone meal compared with yellow corn and barley.

References	Variety	Major Elements				
		Ca	P	Mg	K	Na
[30]	Amhate	0.046	0.085	0.145	0.471	0.025
Yellow corn [31]	-	0.02	0.28	0.12	0.30	0.02
Barley [31]	-	0.03	0.36	0.14	0.48	0.04

**Table 6:** The trace element content (mg/kg of dry matter) of different varieties of date stone meal compared with yellow corn and barley.

References	Variety	Major elements			
		Zn	Cu	Mn	Fe
[30]	Amhate	24.5	5.50	5.7	191
Yellow corn [31]	-	18.0	3.00	7.00	45.0
Barley [31]	-	30.0	10.0	18.0	78.0

**Table 7:** Effects of Date by-products on performance of poultry.

Sources and Levels	Species	Results	Reference
Date pits (0, 10, 20, 30 and 40%),	Broiler chicks	Adding date pits at 10% had no effect on broiler performance. carcass characteristics and blood parameters.	[43]
DW (0 to 200 g/kg diet)	Broiler chicks	No effect on BWG	[36]
In the starting period: DWM (0, 8, 16, 24%) and DPM (5, 10, and 15%) In the finishing period: DWM (0, 8, 16, 24%) and DPM (6, 12 and 18%)	Broiler chicks	In the starting period, BW and BWG were improved when chicks were fed diets containing 8% DWM or 10% DPM, and 10% DPM improved FCR. In the finishing period, chicks fed a diet containing 8% and 16 DWM or 18% DPM had better BW and BWG than the control. 18% DPM improved FCR	[3]
DW (50 and 200 g/kg diet)	Broiler chicks	DW (50 g/kg diet) reduced FI and improved feed efficiency and European feed production efficiency.	[12]
Date pits (5, 10 and 15%) and whole zahdi dates (5, 10 and 47.7%)	Broiler chicks	47.7% whole date as a total replacement of corn resulted in a slight decrease in FCR	[14]

DW = date waste; DWM = date waste meal; DPM = date pits meal; BW = body weight; BWG = body weight gain; FCR = feed conversion ratio; FI = feed intake.

## Data Availability

All data are published in the cited literature and reported in the text of this manuscript.
